# Prevalence and characteristics of cancer patients with COVID-19: a meta-analysis study

**DOI:** 10.12688/f1000research.53539.1

**Published:** 2021-09-27

**Authors:** Johan S. Sitanggang, Kamal B. Siregar, Henry H. Sitanggang, Noverita Sprinse Vinolina

**Affiliations:** 1 Faculty of Medicine, Universitas Sumatera Utara, Medan, Indonesia; 2 Department of Surgery, Oncology Subdivision, Faculty of Medicine, Universitas Sumatera Utara, Medan, Indonesia; 3 Department of Surgery, Head and Neck Oncology Subdivision, Faculty of Medicine, Deli Serdang Hospital, Deli Serdang, Indonesia; 4 Statistics Division, Universitas Sumatera Utara, Medan, Indonesia

**Keywords:** prevalence; COVID-19; cancer; comorbid; severe event; fatality

## Abstract

**Background: **Cancer patients are considered susceptible to coronavirus disease (COVID-19) due to an immunosuppressive state. This study determined the prevalence of cancer in COVID-19 patients, severe events, case fatality rate, history of anticancer therapy associated with severe events, and type of cancer in cancer patients with COVID-19 in the world.

**Methods:** This study used a meta-analysis study approach, sourcing studies from various countries related to cancer and COVID-19. Inclusion and exclusion criteria were established to select studies. A PRISMA flowchart was presented to assess the selection process. Data from inclusion studies were analyzed using Review Manager 5.4.

**Results:** The prevalence of cancer in COVID-19 patients was 4.63% (95% CI, 3.78-5.49%) worldwide. The lowest prevalence was the Asian study group with 2.36% (95% CI, 1.86-2.87%) and the highest prevalence was the European study group with 10.93% (95% CI, 6.62-15.24%). About 43.26% (95% CI, 34.71-51.80%) of cancer patients with COVID-19 experienced severe events of COVID-19. In total, 58.13% (95% CI, 42.79-73.48%) of cancer patients with COVID-19 who in the last month had a history of anticancer therapy experienced severe events. The prevalence of lung cancer in cancer patients with COVID-19 was 20.23% (95% CI, 7.67-32.78%). Forest plots are also presented related to the results of meta-analysis research.

**Conclusions:** High prevalence of cancer among COVID-19 patients indicates the susceptibility of cancer patients to SARS-CoV-2 infection. Cancer in COVID-19 patients and use of anticancer therapy increase severe events of COVID-19.

## Introduction

On December 31, 2019, the World Health Organization (WHO) was notified of cases of pneumonia of unknown cause, which were detected in Wuhan City, Hubei Province, China. From 31 December 2019 to 3 January 2020, a total of 44 pneumonia cases with unknown etiology were reported to the WHO by national authorities in China. The Chinese Centers for Disease Control and Prevention identified a new strain of coronavirus, namely Severe Acute Respiratory Syndrome Coronavirus 2 (SARS-CoV-2) *,* with the name of disease given as Coronavirus Disease 2019 (COVID-19) ^1^.

Confirmed cases of COVID-19 are continually increasing in the world. On January 30, 2020, WHO designated COVID-19 as a Public Health Emergency of International Concern ^2^. Approximately 197,788,117 cumulative cases of COVID-19 had been confirmed and 4,219,578 cumulative deaths had been caused by the COVID-19 disease as of August 3, 2021 ^3^.

The existence of the COVID-19 pandemic also affects and increases various risks in individuals with chronic diseases. Of the 1,590 cases of COVID-19 in 575 hospitals in 31 provinces of China, 399 cases were reported to have comorbid diseases. The most common comorbidity found was hypertension with 269 people (16.9%), followed by cardiovascular and cerebrovascular diseases with 59 (3.7%) and 30 (1.9%), respectively. Meanwhile, cancer was also found in 18 (1.1%) of 1,590 people ^4^. Patients with cancer are more susceptible to infection and may have a higher risk of experiencing a severe event of COVID-19 than individuals without cancer because of their systemic immunosuppressive states caused by malignancies and anticancer treatments, such as chemotherapy or surgery ^5^. The severe event in this study was defined as the condition of patients with severe symptoms, patients admitted to the intensive care unit, patients requiring ventilation, or patient death.

Therefore, the existence of a meta-analysis study which in principle combines the results of research from various countries around the world, could make epidemiological assessments of the prevalence of cancer in COVID-19 patients more accurate. In addition, this meta-analysis study also explained the latest developments based on inclusion studies related to cancer and COVID-19. The prevalence of severe event, death, history of anticancer therapy, and types of cancer in cancer patients with COVID-19 were also included in this study.

Using the PICO (patient, intervention, comparison, and outcome) principle, the patients in this study are COVID-19 patients and, there is no intervention. The comparison found in this study is the history of using anticancer therapy, and the outcome sought is the prevalence of cancer patients in COVID-19 patients, the prevalence of severe event in cancer patient with COVID-19, case fatality rate of cancer patients with COVID-19, and the prevalence of severe event in cancer patient with the history of using anticancer therapy within one month. The PICO question statement that may be obtained is related to the prevalence of cancer patients in COVID-19 patients, what is the prevalence of the incidence of severity of cancer patients with COVID-19, and the prevalence of cancer and COVID-19 patients who experienced severe events with a history of anticancer therapy, especially in the last one month.

## Methods

### Type of research

This research uses a meta-analysis study method to estimate the frequency of clustered diseases, such as prevalence and case fatality rate. The time for conducting the research was four months, from August to November 2020. The total series of processes starting from submitting ethics to accountability for research results at Universitas Sumatera Utara took seven months, from July 2020 to January 2021. The checklist used in this meta-analysis was the PRISMA 2009 Checklist.

### Research sample

The data extraction was carried out using a piloted form with inclusion and exclusion criteria. The inclusion criteria were studies with COVID-19 patient subjects, number and prevalence of COVID-19 patients who also experienced cancer, and journals were in English (pre-print articles and full peer reviewed) that had been circulating on the internet until October 31, 2020. The exclusion criteria of this study were review articles, comments, research conducted on animals, and research that did not contain information regarding the number and prevalence of COVID-19 patients with cancer.

An online literature search was conducted, sourcing from Pubmed, Science Direct, Springerlink, and Google Scholar. Medical subject headings (MeSH) words used to form the search strategy were “prevalence” AND (“cancer” OR “malignancy” OR “tumor”) AND (“COVID-19” OR “coronavirus” OR “SARS-CoV-2”). The data retrieved was the name of the first author, year of publication, data on the number, prevalence, and several characteristics of cancer patients in COVID-19 patients based on research that had been circulating on the internet until October 31, 2020.

There were three reviewers, namely Johan S. Sitanggang, Kamal B. Siregar, and Henry H. Sitanggang, who screened articles for this meta-analysis. Initial screening was carried out by looking at the suitability of the title against the inclusion and exclusion criteria as well as the study abstract. Studies were then assessed in full-text to assess the presence of information related to prevalence that can be retrieved according to the inclusion and exclusion criteria. Information related to the prevalence of cancer patients in COVID-19 patients, the prevalence of cancer patients and COVID-19 who experienced severe events, case fatality rate of cancer patients and COVID-19, prevalence of severe events in cancer patients with a history of using chemotherapy in the last one month, and prevalence of specific cancer (lung cancer) in COVID-19 patients with cancer, was extracted from the full-text study data and recapitulated with table in Review Manager 5.4.

In the process of selecting and extracting information from the original study, the reviewers also looked at the research methodology of the original study, the confidence interval, and the p-value of each related study to assess the potential bias of individual studies. The method used in relation to the risk of bias accross studies in this prevalence meta-analysis is quantitative assessment of the p value and z test of each prevalence data table. Prevalence rate and case fatality rate data processing in this meta-analysis study was determined whether by random effect or fixed effect by assessing I ^2^. If I ^2^ is more than 50%, it indicates high heterogeneity between studies, so the random effects model is used. Meanwhile, if I ^2^ is less than 50%, then the fixed effects model is used. There is no additional analysis other than what has been described previously.

### Processing and analysis of data

Data processing that would be carried out in this study was a meta-analysis study. Prevalence rate (PR), 95% confidence interval (CI) were analyzed using *Review Manager 5.4* software ( *The Cochrane Collaboration*, Oxford, UK). The heterogeneity between the studies was estimated using the I ^2^ test and q.

## Results and discussions

### Literature tracing and selection

**Figure 1.**
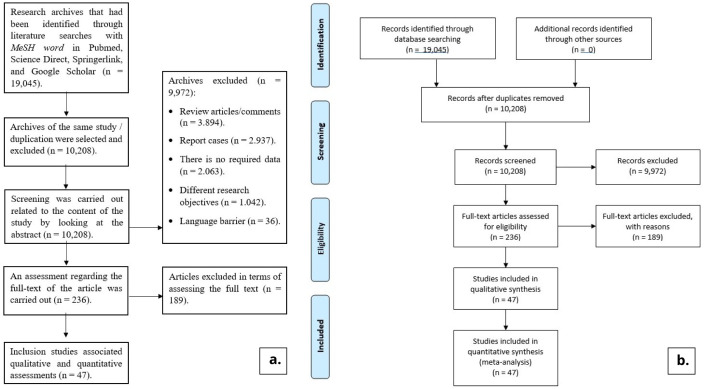
Flowchart ( **a**. detailed literature tracing and selection flowchart, **b**. PRISMA flowchart).

From the results of literature searches up to October 31, 2020 using MeSH words predetermined, 19,045 literatures were found on Google Scholar, Pubmed, Springer Link, and Science Direct. Of the total 19,045 literatures obtained, 16,500 came from a Google Scholar search. Meanwhile, 1,794 literatures came from Science Direct, 558 literatures came from Springer Link, and 193 literatures came from Pubmed. After going through the selection process, in the end, 47 research literatures were included in this study. The process of searching and selecting the literature for this study can be seen in Figure 1.

### Characteristics of the studies

These studies included information regarding the number, prevalence, and characteristics of cancer patients with COVID-19. The characteristics of each study that had been included in this study could be seen in Table 1.

According to the origin of the studies, these studies were found to come from several countries which could be divided based on the location of the country on the continent. The majority of studies that were included in this meta-analysis came from Asia with a total of 33 studies. In this study, there were nine studies (19.1% of all studies) originating from America with all studies originating from the United States of America. Fom Europe, there were five studies (10.6%) included in this meta-analysis.

**Table 1.** Included studies’ characteristics.

Ref.	Study origin	Case identification date	Type of study	Total COVID-19 patients (n)	Patients with cancer (n)	Age of COVID-19 patients (years; median (range))	Gender (Male n (%)/ Female n (%))
Asia							
Cai *et al*., 2020 ^6^	Shenzen, China.	January 11, 2020 – February 6, 2020	Retrospective study, single-center	298	4	47,5 (33 – 61)	145 (48.7) / 153 (51.3)
Cao *et al*., 2020 ^7^	Shanghai, China	January 20, 2020 – February 15, 2020	Cohort study, single- center	198	4	50,1 (33,8 – 66,4)	101 (51) / 97 (49)
Chen, N. *et al*., 2020 ^8^	Wuhan, China	January 1, 2020 – January 20, 2020	Retrospective study, single-center	99	1	55,5 (42,4 – 68,6)	67 (67.7) / 32 (32.3)
Chen, Q. *et al*., 2020 ^9^	Zhejiang, China	January 1, 2020 – March 11, 2020	Retrospective study, single-center	145	3	47,5 (32,9 – 62,1)	79 (54.5) / 66 (45.5)
Chen, T. *et al*., 2020 ^10^	Wuhan, China	January 13, 2020 – February, 12 2020	Retrospective study, single-center	274	7	62 (44 – 70)	171 (62.4) / 103 (37.6)
Chen, T.L. *et al*., 2020 ^11^	Wuhan, China	January 1, 2020 – February 10, 2020	Retrospective study, single-center	203	7	54 (20 – 91)	108 (53.2) / 95 (46.8)
Cheng *et al*., 2020 ^12^	Wuhan, China	January 28, 2020 – February 11, 2020	Cohort study, single-center	698	32	63 (50 – 71)	367 (52.6) / 334 (47.4)
Du *et al*., 2020 ^13^	Wuhan, China	January 9, 2020 – February 15, 2020	Retrospective study, multi-center	85	6	65,8 (51,6 – 80)	62 (72.9) / 23 (27.1)
Feng *et al*., 2020 ^14^	Wuhan, Shanghai, and Anhui, China	January 1, 2020 – February 15, 2020	Retrospective study, multi-center	476	12	53 (40 – 64)	271 (56.9) / 205 (43.1)
Guan *et al*., 2020 ^15^	30 provinces, China	December 11, 2019 – January 29, 2020	Cohort study, multi-center	1099	10	47 (35 – 58)	640 (58.2) / 459 (41.8)
Guo, T. *et al*., 2020 ^16^	Wuhan, China	January 23, 2020 – February 23, 2020	Retrospective study, single-center	187	13	58,5 (43,84 – 73,16)	91 (48.7) / 96 (51.3)
Guo, W. *et al*., 2020 ^17^	Wuhan, China	February 10, 2020 – February 29, 2020	Retrospective study, single-center	174	17	59 (49 – 67)	76 (43.7) / 98 (56.3)
Huang *et al*., 2020 ^18^	Wuhan, China	December 16, 2019 – January 2, 2020	Cohort study, single-center	41	1	49 (41 – 58)	30 (73.2) / 11 (26.8)
Lian *et al*., 2020 ^19^	Zhejiang, China	January 17, 2020 – February 12, 2020	Retrospective study, single-center	788	6	≥ 60 group: 68,28 (60,966 – 75,594) < 60 group: 41,15 (29,77 – 52,53)	407 (51.6) / 381 (48.4)
Liang *et al*., 2020 ^5^	31 provinces, China	January 31, 2020	Cohort study, multi- center	1590	18	Cancer and COVID-19 patients’ age: 63,1 (51 – 75,2); COVID-19 patients’ age without cancer: 48,7 (32,5 – 64,9)	Not available
Liu, K. *et al*., 2020 ^20^	Hubei Province, China	December 30, 2019 – January 24, 2020	Retrospective study, multi-center	137	2	57 (20 – 83)	61 (44.5) / 76 (55.5)
Ma *et al*, 2020 ^21^	Wuhan, China	January 1, 2020 – March 30, 2020	Retrospective study, single-center	1380	37	Cancer and COVID-19 patients’ age: 62 (59 – 70)	20 (54.1) / 17 (45.9) (Cancer and COVID-19 patients’ gender ratio)
Mo *et al*., 2020 ^22^	Wuhan, China	January 1, 2020 – February 5, 2020	Retrospective study, single-center	155	7	54 (42 – 66)	86 (55.5) / 69 (44.5)
Shi *et al*., 2020 ^23^	Wuhan, China	December 20, 2019 – January 23, 2020	Retrospective study, multi-center	81	4	49,5 (38,5 – 60,5)	42 (51.9) / 39 (48.1)
Wan *et al*., 2020 ^24^	North East Chongqing, China	January 23, 2020 – February 8, 2020	Retrospective study, single-center	135	4	47 (36 – 55)	72 (53.3) / 63 (46.7)
Wang, D. *et al*., 2020 ^25^	Wuhan, China	January 1, 2020 – January 28, 2020	Retrospective study, single-center	138	10	56 (42 – 68)	75 (54.3) / 63 (45.7)
Wu *et al*., 2020 ^26^	Wuhan, China	December 25, 2019 – January 26, 2020	Retrospective study, single-center	201	1	51 (43 – 60)	128 (63.7) / 73 (36.3)
Yang *et al*., 2020 ^27^	Wuhan, China	December 31, 2019 – January 26, 2020	Retrospective study, single-center	52	2	59,7 (46,4 – 73)	35 (67.3) / 17 (32.7)
Zhang, G. *et al*., 2020 ^28^	Wuhan, China	January 2, 2020 – February 10, 2020	Retrospective study, single-center	221	9	55 (39 – 66,5)	108 (48.9) / 113 (51.1)
Zhang, J. *et al*., 2020 ^29^	Wuhan, China	January 13, 2020 – February 16, 2020	Retrospective study, single-center	111	8	38 (32 – 57)	46 (41.4) / 65 (58.6)
Zhang, L. *et al*., 2020 ^30^	Wuhan, China	January 13, 2020 – February 26, 2020	Retrospective study, multi-center	1276	28	Cancer and COVID-19 patients’ age: 65 (56 – 70)	17 (60.7) / 11 (39.3) (Cancer and COVID-19 patients’ gender ratio)
Zhou *et al*., 2020 ^31^	Wuhan, China	December 29, 2019 – January 31, 2020	Retrospective study, multi-center	191	2	56 (46 – 67)	119 (62.3) / 72 (37.7)
Zhu *et al*., 2020 ^32^	Hefei, Anhui Province, China	January 24, 2020 – February 20, 2020	Retrospective study, multi-center	32	2	46 (35 – 52)	15 (46.9) / 17 (53.1)
Jeong *et al*., 2020 ^33^	South Korea	March 12, 2020	Retrospective study, multi-center	66	7	77 (35 – 93)	37 (56.1) / 29 (43.9)
Kang *et al*., 2020 ^34^	South Korea	March 16, 2020	Retrospective study, multi-center	75	10	Not available	Not available
Kim, E.S. *et al*., 2020 ^35^	South Korea	January 19, 2020 – February 17, 2020	Cohort study, multi-center	28	1	42,6 (29,2 – 56)	15 (53.6) / 13 (46.4)
Tabata *et al*., 2020 ^36^	Tokyo, Japan	February 11, 2020 – February 25, 2020	Retrospective study, single-center	104	4	68 (46,75 – 75)	54 (51.9) / 50 (48.1)
Nikpour-aghdam *et al*., 2020 ^37^	Tehran, Iran	February 19, 2020 – April 15, 2020	Retrospective study, single-center	2964	17	56 (46 – 65)	1955 (65.9) / 1009 (34.1)
Americas							
Argenzi-ano *et al*., 2020 ^38^	New York, USA	March 1, 2020 – April 5, 2020	Retrospective study, single-center	1000	67	63 (50 – 75)	596 (59.6) / 404 (40.4)
Cummings *et al*., 2020 ^39^	New York, USA	March 2, 2020 – April 1, 2020	Cohort study, multi-center	257	18	62 (51 – 72)	171 (66.5) / 86 (33.5)
McMichael *et al*., 2020 ^40^	Washington, USA	February 27, 2020 – March 18, 2020	Retrospective study, single-center	167	15	72 (21 – 100)	55 (32.9) / 112 (67.1)
Miyashita *et al*., 2020 ^41^	New York, USA	March 1, 2020 – April 6, 2020	Cohort study, single-center	5688	334	Not available	Not available
Myers *et al*., 2020 ^42^	California, USA	March 1, 2020 – March 31, 2020	Retrospective study, multi-center	377	18	61 (50 – 73)	212 (56.2) / 165 (43.8)
Petrilli *et al*., 2020 ^43^	New York, USA	March 1, 2020 – April 2, 2020	Cross-sectional study, single-center	1582	110	62,5 (46 – 77)	1002 (63.3) / 580 (36.7)
Paranjpe *et al*., 2020 ^44^	New York, USA	February 27, 2020 – April 2, 2020	Descriptive study, multi-center	2199	151	65 (54 – 76)	1293 (58.8) / 906 (41.2)
Rentsch *et al*., 2020 ^45^	USA	February 8, 2020 – March 30, 2020	Retrospective study, multi-center	585	83	66,1 (60,4 – 71)	558 (95.4) / 27 (4.6)
Richard-son *et al*., 2020 ^46^	New York, USA	March 1, 2020 – April 4, 2020	Retrospective study, multi-center	5700	320	63 (52 – 75)	3437 (60.3) / 2263 (39.7)
Europe							
Benelli *et al*., 2020 ^47^	Crema, Italy	February 21, 2020 – March 13, 2020	Cohort study, single-center	411	33	66,8 (50,4 – 83,2)	274 (66.6) / 137 (33.4)
Colaneri *et al*., 2020 ^48^	Pavia, Italy	February 21, 2020 – February 28, 2020	Cohort study, single-center	44	6	67,5 (52,95 – 82,05)	28 (63.6) / 16 (36.4)
Grasselli *et al*., 2020 ^49^	Milan, Italy	February 20, 2020 – March 18, 2020	Retrospective study, multi-center	1591	81	63 (56 – 70)	1304 (81.9) / 287 (18.1)
Rossi *et al*., 2020 ^50^	Reggio Emilia, Italy	February 27, 2020 – April 2, 2020	Cohort study, multi-center	2653	301	Not available	1328 (50.1) / 1325 (49.9)
Lovell *et al*., 2020 ^51^	London, England	March 4, 2020 – March 26, 2020	Retrospective study, multi-center	101	25	82 (72 – 89)	64 (63.4) / 37 (36.6)

## Meta-analysis results of cancer prevalence in COVID-19 patients

### Prevalence by continent area

**Figure 2.**
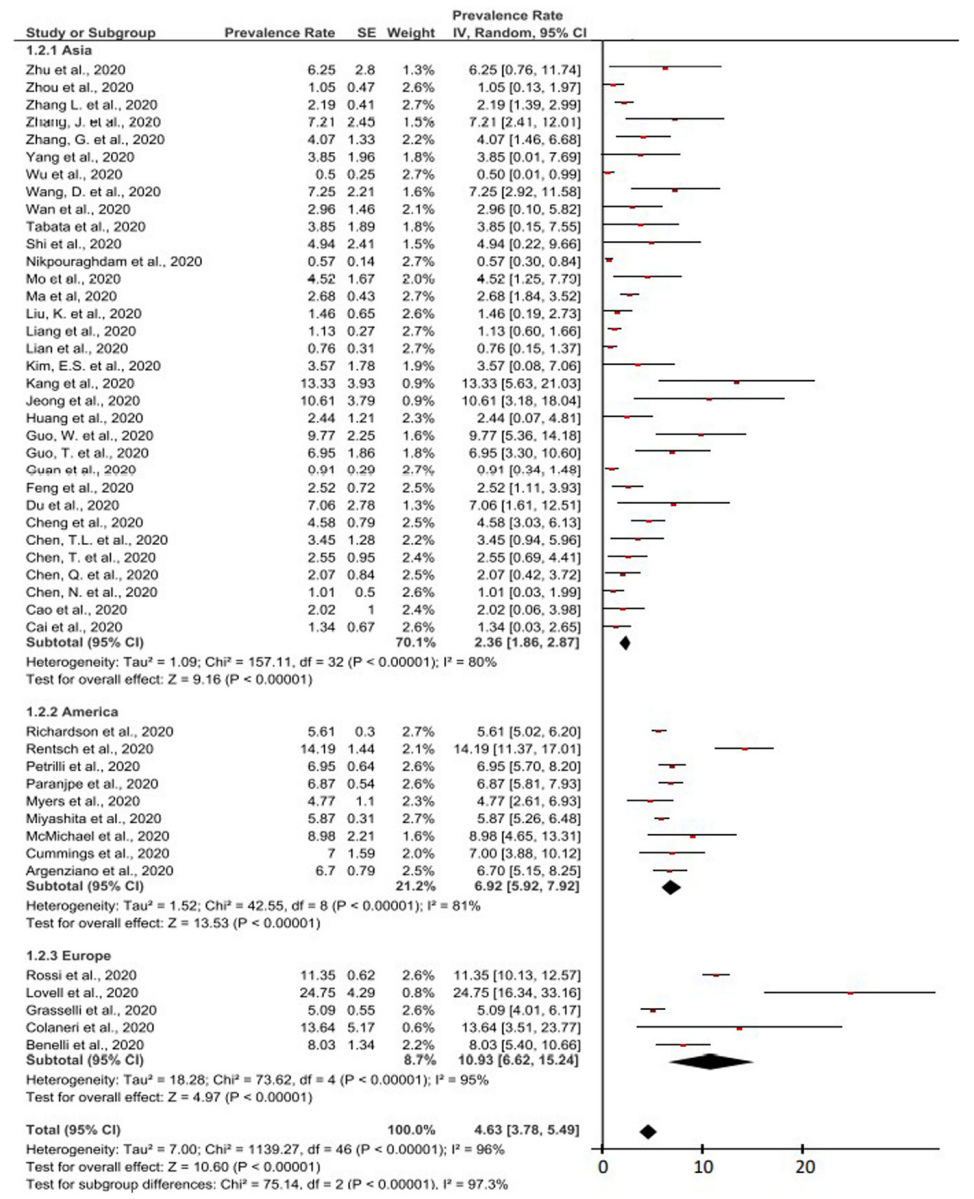
Forest plot of the prevalence of cancer in COVID-19 patients by continent.

In Figure 2, a forest plot for a total of 47 studies that had been included from various regions of the world. Based on this meta-analysis, it had been found that the overall prevalence of cancer patients in COVID-19 patients was 4.63% (95% CI, 3.78-5.49%). As for the heterogeneity test in this meta-analysis, it had been found that the I ^2^ value was 96% (>75%). This indicates a high degree of heterogeneity in the overall study results. Therefore, a meta-analysis was performed with random effect (>50%). High heterogeneity was also indicated by the P value <0.0001 (<0.05) in this study. The result of the P value on the Z-test was <0.0001 (<0.05), which means that the 47 studies’ data had significant and important values.

Therefore, the prevalence of cancer in COVID-19 patients in the world had been found to be eight times higher than the prevalence value of cancer in the whole world population based on the latest WHO data. The prevalence of cancer sufferers in the world community only reaches 0.57% ^52^. The high prevalence of cancer patients in COVID-19 patients shows that cancer sufferers are more susceptible to infection from the SARS-CoV-2 virus, which must be closely monitored.

On the Asian continent, the results of the meta-analysis of the prevalence of cancer patients in COVID-19 patients was 2.36% (95% CI, 1.86-2.87%). Meanwhile, in the Americas, the results of the meta-analysis of the prevalence of cancer patients in COVID-19 patients was 6.92% (95% CI, 5.92-7.92%).

Based on studies originating from Europe, the results of the meta-analysis of the prevalence of cancer in COVID-19 patients was 10.93% (95% CI, 6.62-15.24%). The prevalence of cancer patients in COVID-19 patients in Europe was the highest compared to the prevalence of the two other continents.

### Severe event and death in cancer patients with COVID-19

According to the studies that had been included, there were reports of cancer patients with COVID-19 experiencing a severe event, and case deaths. The severe event in this study was defined as the condition of patients with severe symptoms, patients admitted to the intensive care unit, patients requiring ventilation, or even death.

**Figure 3.**
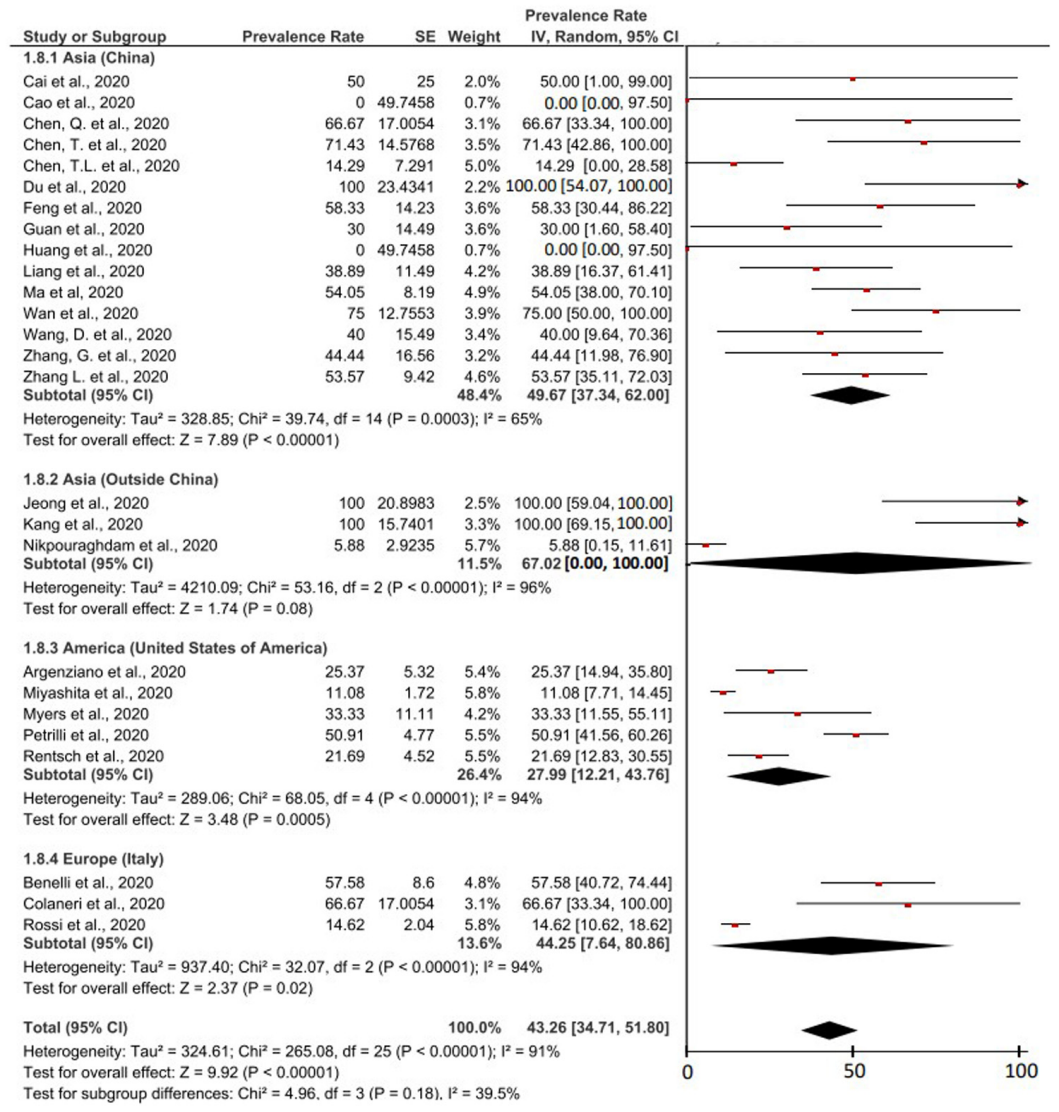
Forest plot of prevalence of severe event in cancer patients with COVID-19.

Figure 3 presents the prevalence of severe event that occurs in cancer patients with COVID-19. Based on meta-analysis calculations from a total of 26 studies containing information regarding severe event in cancer patients with COVID-19, it was found that the prevalence value was 43.26% (95% CI, 34.71-51.80%). The I ^2^ value was 91% (>75%), so that the calculation of this meta-analysis also used random effect. Heterogeneous P values and P on the Z-test were found to be <0.00001 (<0.05) which was heterogeneous and significant.

Based on the studies originating from China, it was found that the prevalence of severe event in cancer patients with COVID-19 was 49.67% (95% CI, 37.34-62.00%). Meanwhile, according to three other studies originating from Asia outside China, it was found that 67.02% (95% CI, 0.00-100.00%) of cancer patients with COVID-19 experienced severe event. The prevalence of severe event based on studies originating from Asia outside of China had the highest prevalence value among other groups ^33, 34, 37^.

The prevalence of severe event based on American studies was the lowest of the other groups. Based on 5 studies from America, 27.99% (95% CI, 12.21-43.76%) cancer patients with COVID-19 experienced severe event. Additionally, it was found that 44.25% (95% CI, 7.64-80.86%) of European cancer patients with COVID-19 experienced severe event.

**Figure 4.**
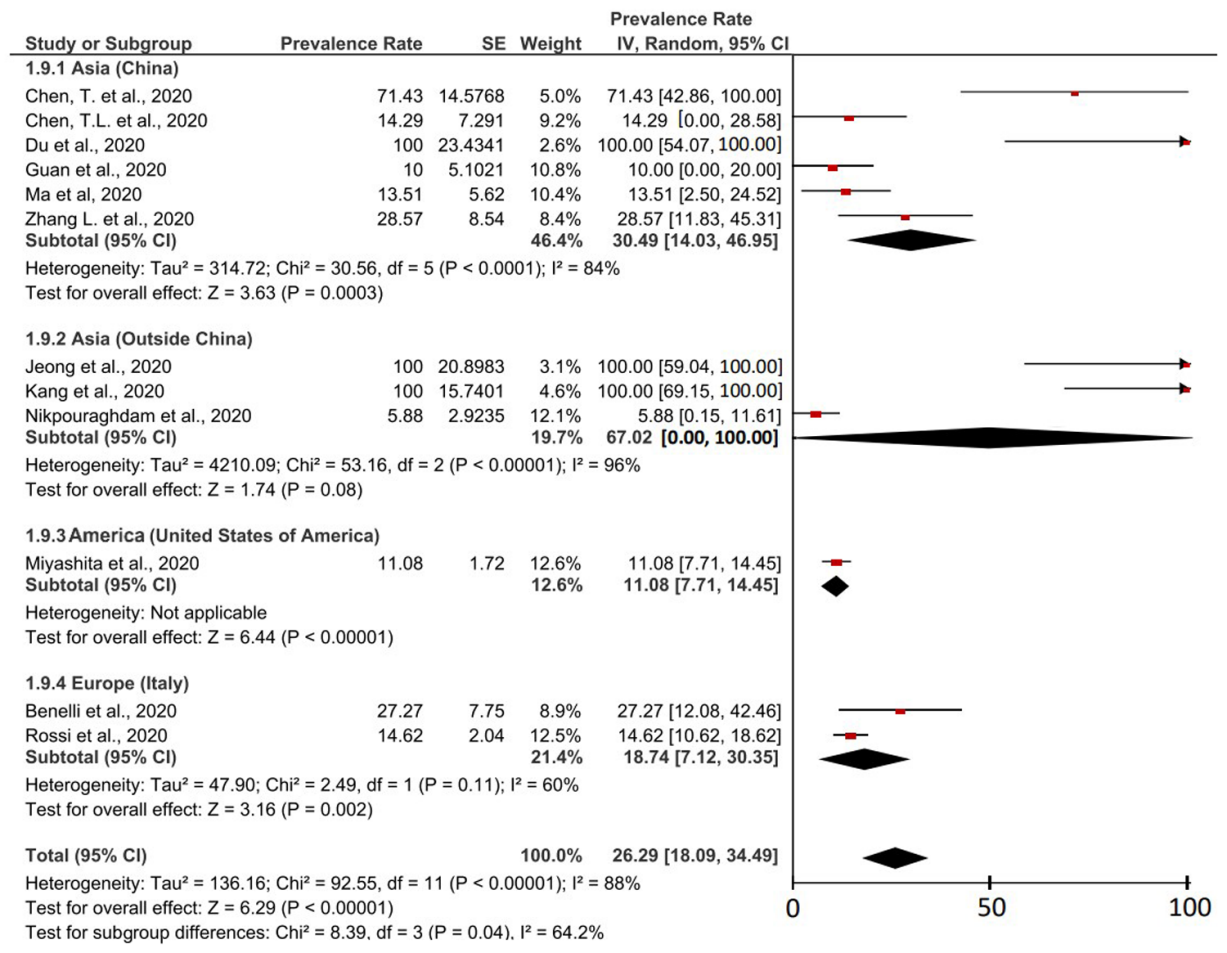
Forest plot of case-fatality rates in cancer patients with COVID-19.

The fatality rate of COVID-19 cases in cancer patients was based on 12 studies which were described in detail in Figure 4. The result was a case fatality rate of 26.29% (95% CI, 18.09-34.49%) of cancer patients with COVID-19 who experienced death. Based on the I ^2^ value related to the heterogeneity of the study, it was found that a high level of heterogeneity was obtained with a I ^2^ of 88%. Therefore, the principle of random effect was used in calculating the prevalence meta-analysis. Heterogeneous P values and P on the Z-test were found <0.00001 (<0.05) which was heterogeneous and significant.

### History of anticancer therapy for cancer patients with COVID-19

**Figure 5.**
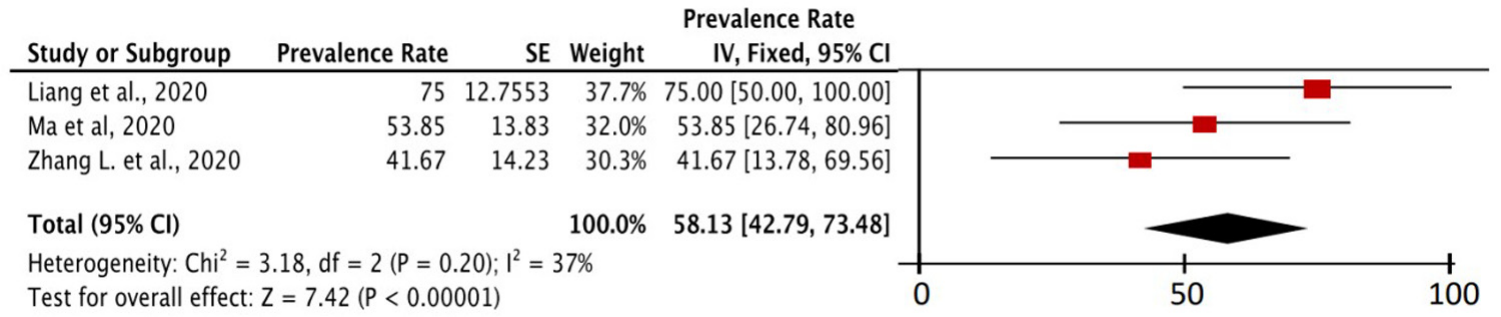
Forest plot of prevalence of severe event of cancer patients due to COVID-19 with a history of anticancer therapy.

In Figure 5, the prevalence of severe event in cancer patients with COVID-19 with a history of anticancer therapy was described at least in the last month. There were three studies that specifically contained this data. Based on meta-analysis calculations from the three studies, 58.13% (95% CI, 42.79-73.48%) of cancer patients with COVID-19 who in the last month at least had a history of anticancer therapy experienced severe event. The P value on the Z-test was found to be <0.00001, which means that the calculation remained significant and important. In addition to exposure and mobility factors in cancer patients, the state of immunosuppression caused by anticancer therapy in cancer patients is also considered an important factor of susceptibility to COVID-19. The prevalence of the COVID-19 severe event in patients with cancer who had a history of anticancer therapy in the last month was 1.34 times higher than the prevalence of severe event of COVID-19 in cancer patients as a whole.

### Types of cancer in cancer patients with COVID-19

**Figure 6.**
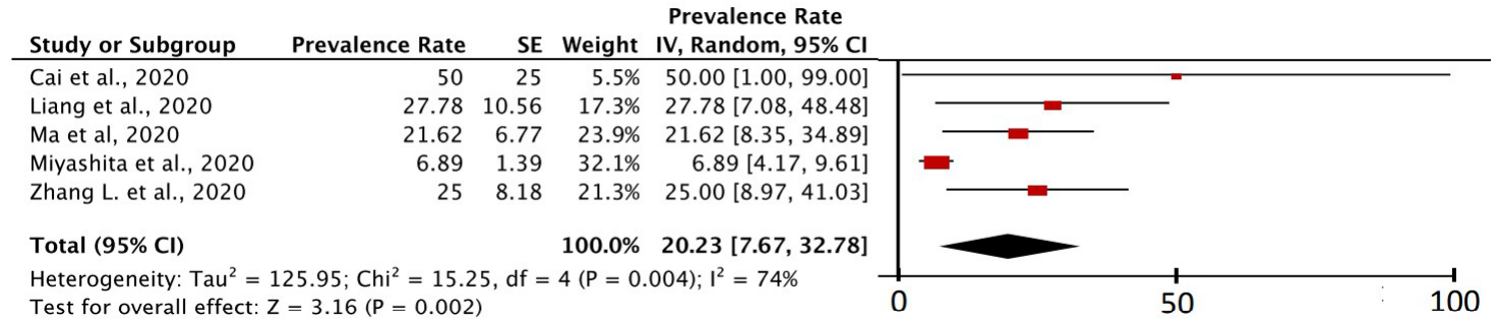
Forest plot of lung cancer prevalence in cancer patients with COVID-19.

Figure 6 shows that from five studies that specifically described data on the types of cancer found in cancer patients with COVID-19, lung cancer was found in five studies. The value I ^2^ of the five studies was 74%, which was greater than 50%. So, the principle of random effect was used in the calculation. The P value of heterogeneity was found to be 0.004 (<0.05) and the P value of the *Z-test* was 0.002 (<0.05), which means that the study data from this calculation was heterogeneous and significant.

## Discussion

This meta-analysis study covered a large area and representative as prevalence and epidemiological data related to cancer and COVID-19, which can be seen in Figure 2. The overall prevalence of cancer patients in COVID-19 patients in the world was 4.63% (95% CI, 3.78% - 5.49%). The highest prevalence of cancer in COVID-19 patients by continent area was found in Europe with 10.93% (95% CI, 6.62% - 15.24%). The highest prevalence of cancer in COVID-19 patients reported by a single study came from the UK, with 24.75% (95% CI, 16.34% – 33.16%) ^51^. Meanwhile, the lowest prevalence was Asia 2.36% (95% CI, 1.86-2.87%). The lowest prevalence by a single study was obtained with a prevalence of 0.50% (0.01% – 0.99%), which was taken from a study from China ^26^.

In the calculation of the meta-analysis of 47 studies, the I ^2^ value, the P value of heterogeneity, and the P value of the Z-test were also presented. The heterogeneity test of the I ^2^ value in this meta-analysis was found to be 96% (>75%). This indicates that the data from the 47 studies has a high level of heterogeneity. The P value of heterogeneity was also found to be <0.0001 (<0.05) which indicates a high level of heterogeneity and significancy. The P value of the Z-test in this forest plot is <0.0001 (<0.05), which means that 47 studies’ data have significant and important values. The high level of heterogeneity and significance value in this meta-analysis calculation can prove that there is no possibility of bias from the authors on the results of the meta-analysis calculations. In addition, all meta-analysis calculations in this study were found to be meaningful or significant in the results.

Cancer in COVID-19 patients and use of anticancer therapy affect severe events of COVID-19 patients. Based on inclusion studies that specifically describe the history of anticancer therapy in cancer patients with COVID-19, we found 58.13% (95% CI, 42.79% – 73.48%) of cancer patients who has COVID-19 and a history of anticancer therapy, experienced a serious event (in Figure 5). This prevalence value is 1.34 times higher than the overall prevalence value of severe event in cancer patients with COVID-19 (43.26%, 95% CI, 34.71% – 51.80%) which can be seen in Figure 3.

Specifically, this study also includes a meta-analysis study related to the prevalence of certain types of cancer, namely lung cancer (in Figure 6). This is because in several inclusion studies, lung cancer was mentioned as the type of cancer with the highest prevalence compared to other cancers in COVID-19 patients in their study research samples ^5, 6, 21, 30, 41^. Based on the calculation of the prevalence of lung cancer in cancer patients with COVID-19, it was found that 20.23% (95% CI, 7.67% – 32.78%) of cancer patients with COVID-19 were lung cancer patients. The I ^2^ value of the five studies was 74%, which was greater than 50%. Indeed, based on these results, the heterogeneity level of the inclusion study was not at the highest level of heterogeneity, but the results were heterogeneous enough to make this meta-analysis calculation using random effects. The P value of heterogeneity was found to be 0.004 (<0.05) and the P value of the Z-test was 0.002 (<0.05), which means that the study data from this calculation was heterogeneous and significant.

The high prevalence of COVID-19 severe event in cancer patients with a history of anticancer therapy means that anticancer therapy is an important factor in the occurrence of poor outcomes in cancer patients with COVID-19. Therefore, cancer patients who are about to undergo anticancer therapy must be closely monitored so they are not exposed to SARS-CoV-2. In patients with suspected symptoms of COVID-19, it is advisable to consider delaying some anticancer therapies such as chemoterapy, surgery, radiotherapy, and others.

Aside from the results reported above, there are several obstacles and shortcomings found in the work of this study. In determining a criteria for a severe event, until now there is still no specific value or scoring criteria that determines how severe a patient's condition is caused by COVID-19.

High prevalence of cancer among COVID-19 patients indicates the susceptibility of cancer patients to SARS-CoV-2 infection. Cancer in COVID-19 patients and use of anticancer therapy affect the prevalence of a severe event of COVID-19 patients. The prevalence of severe event in patients with cancer and COVID-19 who had a history of anticancer therapy in the last 1 month was 1.34 times higher than the prevalence of severe event in cancer patients with COVID-19 as a whole. This means that a history of anticancer therapy may influence the occurrence of COVID-19 severity in cancer patients with COVID-19. All authors hope that more specific research about COVID-19 and certain type of cancer in the future will be carried out.

## Data availability

### Underlying data

All data underlying the results are available as part of the article and no additional source data are required.

### Reporting guidelines

figshare: PRISMA checklist for ‘Prevalence and characteristics of cancer patients with covid-19: a meta-analysis study’. https://doi.org/10.6084/m9.figshare.16590044 ^53^

Data are available under the terms of the Creative Commons Attribution 4.0 International license (CC-BY 4.0).

## Ethical approval

Based on the approval of the health research implementation ethics committee No. 462 / KEP / USU / 2020, Chair of the Research Ethics Committee of the Universitas Sumatera Utara, after carrying out discussion and assessment of research proposals based on the rules of the Neuremberg Code and the Declaration of Helsinki, decided on a study entitled, "Prevalence and Characteristics of Cancer Patients with COVID-19: a Meta-Analysis Study", approved for implementation.
