# Supplementary material for: Prevalence of cancer as a comorbid in COVID-19 patients and their characteristics: a meta-analysis study
Source: F1000Res. 2022 Jan 27;10:975. Originally published 2021 Sep 27. [Version 2] doi: 10.12688/f1000research.53539.2 (PMC9382154; doi:10.12688/f1000research.53539.2)
Supplement: Peer review history

# Peer review: Reviewer response for version 2

**DOI:** 10.5256/f1000research.79084.r145517

**Published:** 2022-08-16

**Authors:** Patumrat Sripan

**Affiliations:** 1 Research Institute for Health Sciences, Chiang Mai University, Chiang Mai, Thailand

This study used the meta-analysis approach to describe the prevalence of cancer as a comorbid in COVID-19 patients, severe events, case fatality rate, history of anticancer therapy associated with severe events, and type of cancer in cancer patients with COVID-19. 

 I have a few comments as follows.

1. In the conclusion, the authors pointed out that the use of anticancer therapy may increase the prevalence of severe events in COVID-19 patients. I found this interesting. It can raise the related research questions for a specific type of research study design to confirm this result. However, this conclusion is based on the evidence from only 3 studies in China. The authors should comment that this conclusion would be stronger with a larger number of publications from various areas including outside of China. 
2. The number of sample sizes in each study should be provided in Figures 2-6 where the prevalence was estimated so that the reader can consider how accurate the estimation is.  

Are the rationale for, and objectives of, the Systematic Review clearly stated?

Yes

Is the statistical analysis and its interpretation appropriate?

Yes

Are sufficient details of the methods and analysis provided to allow replication by others?

Yes

Are the conclusions drawn adequately supported by the results presented in the review?

Partly

Reviewer Expertise:

Public health and biostatistics

I confirm that I have read this submission and believe that I have an appropriate level of expertise to confirm that it is of an acceptable scientific standard.

# Peer review: Reviewer response for version 2

**DOI:** 10.5256/f1000research.79084.r121262

**Published:** 2022-06-28

**Authors:** Tjakra Wibawa Manuaba

**Affiliations:** 1 Division of Surgical Oncology, Department of Surgery, Faculty of Medicine, Udayana University, Denpasar, Indonesia

I have read the recent revision and the comment made by the authors. I have no further questions and this article, in my opinion, is good enough to be indexed.

Are the rationale for, and objectives of, the Systematic Review clearly stated?

Yes

Is the statistical analysis and its interpretation appropriate?

Yes

Are sufficient details of the methods and analysis provided to allow replication by others?

Yes

Are the conclusions drawn adequately supported by the results presented in the review?

Partly

Reviewer Expertise:

NA

I confirm that I have read this submission and believe that I have an appropriate level of expertise to confirm that it is of an acceptable scientific standard.

# Response

**Published:** 2022-07-02

**Authors:** Noverita Sprinse

**Affiliations:** Universitas Sumatera Utara, Indonesia

Thank you for all the suggestions and comments that have been given by the peer reviewer. All inputs and suggestions that have previously been given by reviewer are very useful in the development of this article.

# Peer review: Reviewer response for version 1

**DOI:** 10.5256/f1000research.56935.r96847

**Published:** 2021-11-01

**Authors:** Tjakra Wibawa Manuaba

**Affiliations:** 1 Division of Surgical Oncology, Department of Surgery, Faculty of Medicine, Udayana University, Denpasar, Indonesia

There is only one thing I want to clear up, which is the understanding of the title? Prevalence and characteristics of cancer patients with COVID-19? shouldn’t it be the prevalence of covid-19 in cancer patients and their characteristics? Because looking at the study design - this study is to determine the prevalence of cancer in covid-19 patients, it seems to me that COVID-19 was somehow causing cancer. 

 When actually we were looking at how many cancer patients were infected by covid-19 and how they reacted to the infection and whether certain types of cancer and their treatments influenced the outcomes of covid-19 infection. We understand "the sample finding" was looking at patients with COVID-19 infection, and looked from those patients who had cancer, so this created a thought that COVID-19 was directly correlated to cancer in fact was not, they were cancer patients who happened to be infected by COVID-19.  

 In general, I feel that this article was good and worth indexing.

 Since I am not a biostatistician and this is a meta-analysis study using available data from other studies.  One thing you have to look for is the heterogeneity of the data used. The data have been analysed, and data heterogeneity is not significant (?), so the differences in the study variables are really due to the research hypothesis. 

Are the rationale for, and objectives of, the Systematic Review clearly stated?

Yes

Is the statistical analysis and its interpretation appropriate?

Yes

Are sufficient details of the methods and analysis provided to allow replication by others?

Yes

Are the conclusions drawn adequately supported by the results presented in the review?

Partly

Reviewer Expertise:

NA

I confirm that I have read this submission and believe that I have an appropriate level of expertise to confirm that it is of an acceptable scientific standard, however I have significant reservations, as outlined above.

# Response

**Published:** 2021-11-02

**Authors:** Noverita Sprinse

**Affiliations:** Universitas Sumatera Utara, Indonesia

Dear reviewer,

 Thank you for the review that had been given to this meta-analysis article. We will immediately improve some of the points that have been described by reviewer as follows.

 The first is related to the title, which was originally "Prevalence and Characteristics of Cancer Patients with COVID-19" to "The Prevalence of COVID-19 in Cancer Patients and Their Characteristics". Indeed, our current title may imply the assumption that COVID-19 infection may cause cancer. Therefore, based on suggestions and reviews from reviewer, we made the decision to change the title to "Prevalence of Cancer as a Comorbid in COVID-19 Patients and Their Characteristics". In our opinion, as the authors, the title "COVID-19 Prevalence in Cancer Patients" does not really suitable with the results of the study because the results and sources of the studies used is the prevalence of cancer as a comorbid in COVID-19 patients, which affects the severity of the patients' COVID-19 conditions.

 Then, regarding the heterogeneity and significance of each data, we have conducted heterogeneity and significance tests for each meta-analytical calculation and studies included in this article. In each meta-analysis calculation and included studies, we use the p-value test, Z-score test, and also the I ^2^ test with the results showing heterogeneous and significant, which we have included the results in the discussions column and can also be seen directly in the meta-analysis calculation figure at the bottom left.

 Thank you for the time and thoughts that have been given by reviewer to review this article.

 Best regards,

 Authors
